# Effects of Modified Processing Methods on Structural Changes of Black Soybean Protein Isolate

**DOI:** 10.3390/molecules23092127

**Published:** 2018-08-23

**Authors:** Yinglei Zhang, Yanyang Yin, Shuwen Lu, Xinmiao Yao, Xianzhe Zheng, Rui Zhao, Zhebin Li, Huifang Shen, Shouwen Zhang

**Affiliations:** 1 School of Food Engineering, Harbin University of Commerce, Harbin 150076, China; susanp171221@163.com; 2 Food Processing Institute, Heilongjiang Academy of Agricultural Sciences, Harbin 150086, China; shuwenl@sina.com (S.L.); cocoyococo@163.com (X.Y.); lilyamongthorns@163.com (R.Z.); ienknight@163.com (Z.L.); shenhuifang_1987@126.com (H.S.); 3 Academy of Quality Inspection and Research in Heilongjiang Province, Harbin 150050, China; yanyangtian5092@163.com; 4 College of Engineering, Northeast Agricultural University, Harbin 150030, China; Zhengxz@neau.edu.cn

**Keywords:** black soybean protein isolate, transglutaminase (TG), Maillard reaction, modification, structure

## Abstract

To explore better methods of natural protein modification for black soybean, comparisons among the effects of different modified methods on structural changes of the modified products of black soybean protein isolate (BSPI) were carried out in this study. The modified products used in this study included enzymatic crossing-link black soybean protein isolate (ECBSPI), wet heating treatment glycosylation black soybean protein isolate (WHTGBSPI) and especially enzymatic glycosylation black soybean protein isolate catalyzed by transglutaminase (EGBSPI). The effects of the modification methods on structural changes were analyzed by SDS-polyacrylamide gel electrophoresis (SDS-PAGE), amino acid content and circular dichroism (CD) analysis. Moreover, the processing properties changes caused by structural changes of BSPI were detected by thermogravimetric analysis, particle size analysis, zeta-potential, surface hydrophobicity, solubility, emulsification, gelation, and rheological properties. The results show that the modified BSPI products were protein polymers, and among them, EGBSP and WHTGBSPI are covalently bonded glycation products. Products modified by Maillard reactions and transglutaminase (TG) display partly destroyed α-helix and β-sheet structures that form more open secondary BSPI structures. For ECBSPI, the proportion of irregular crimp structure reduces to form a high order secondary structure. All the modified products form fine aggregations in dispersion, except WHTGBSPI has most negative zeta-potential and least molecular stability due to the hydrophobic amino acids embedded in the protein molecules. The zeta-potentials of BSPI, ECBSPI, WHTGBSPI and EGBSPI are respectively −21.5, −23.8, −18.1 and −20.2 mV. The surface hydrophobicity of EGBSPI (5.07 ± 0.07) and WHTGBSPI (7.02 ± 0.05) decrease, while the surface hydrophobicity of ECBSPI (19.5 ± 0.06) increases. The solubility and rheological properties of EGBSPI, ECBSPI and WHTGBSPI after modification are all better than those of BSPI, especially EGBSPI. Emulsification of EGBSPI and WHTGBSPI increase (by 24.5% and 12.2%, respectively) while ECBSPI decrease (by 17.0), and there is similar emulsion stability trend. Moreover, the properties of ECBSPI increase except cohesiveness compared to BSPI. In conclusion, as a safe and efficient method for natural protein modification, enzymatic glycosylation catalyzed by TG has great potential in improving food processing characteristics.

## 1. Introduction

Black soybean (*Glycine max*), a soybean with black seeds, is widely planted in China, especially in the northeastern area [1]. Black soybean is rich in protein [2], with an obviously higher content of protein and amino acids than that of common soybean [3]. Because of its high contents of hydrophobic amino acids, black bean protein isolate displays excellent emulsifying properties and antioxidant activity, and has become an important emulsifying agent in the food industry. What’s more, the results of our preliminary experiments showed that the 11S globulin amount of BSPI is more than that of soybean protein isolate, which indicates a strong ability to maintain the colloidal network structure via disulfide bonds, hydrophobic interaction forces and complex bonds. However, the ability of 7S globulin-rich protein to maintain a colloidal reticular structure is relatively weak due to hydrophobic interactions and hydrogen bonds. The 11S globulin is also considered to be better as a substrate of transglutaminase (TG) [4]. These make black beans an attractive source of proteins for extraction and modification.

However, as a kind of single source natural protein, not all processing properities of black soybean protein are remarkable. For instance, the solubility of BSPI is not so good. These functional properties could be improved via various modification methods, such as enzymolysis, ultrasound irradiation, glycosylation and so on. There are many reported studies concerned with natural protein modification methods. Li et al. found that rice protein hydrolysates have lower surface hydrophobicity after Maillard reactions [5], but traditional modification methods still have disadvantages in some aspects.

Glycosylation modification of food proteins introduces hydrophilic carbohydrates into food protein molecules by covalent bonding, so that the modified products have both the macromolecular properties of proteins and the hydrophilic properties of carbohydrates. It has been found that soybean protein isolate glycoslated by enzymic methods showed superior emulsifying ability and had different degrees of improvement in solubility, gelation, rheological properties, thermal stability, and antibacterial properties [6]. The efficiency of Maillard reactions is non-ideal due to the very severe reaction conditions. It has been reported that some of these products may cause oxidative stress and neurotoxicity, cause inflammation [7] and promote connective tissue aging [8]. Certain reactions produce furfural that induces mutagenesis and damages genes in mammalian cells. Therefore, the glycosylation of proteins catalyzed by biological enzymes can overcome some deficiencies of the Maillard reaction, and this area has become a new focus in recent years. In theory, there are three enzymes that could be used to catalyze this reaction: glycosyltransferases, glycosidases and transglutaminases (TG). Among them, it is very difficult to get large quantities of glycosyltransferases as commercial forms and very specific, and for glycosidases, there are not many glycosylated products that are synthesized. TG seems to be a good choice for its industrial processing and substrate diversity.

For protein substrates, TG catalyzes the two nucleophilic substitution reactions: (A) it produces γ-glutamyl-ε-lysine side-chain peptides which leads to protein crossinglinking; (B) it introduces small molecule amines into protein substrates. There is competition between the occurrence of these two reactions [9]. Compared with the studies focused on protein crossing-linking catalyzed by TG, only a few studies have reported on protein glycosylation catalyzed by TG. Yan et al. first clarified the feasibility of using TG in catalyzing the glycosylation of proteins [10]. Villalonga et al. used TG to catalyse the glycosylation of β-cyclodextrin with trypsin, and the thermal stability of the glycosyl products was significantly enhanced [11]. Rare studies have been found to focus on the comparison among glycosylation of proteins catalyzed by TG and other modification methods.

In our study, we focused on comparison among different modified methods’ effect (crossing-linking, enzymatic glycosylation and glycoslation by Maillard reactions) on BSPI. Based on this, there were more investigations about structural changes of soybean protein after modifcation. Key property changes of the modified proteins were characterized as well, which provided a theoretical basis for further selection and application of different soybean protein modification methods in the food processing industry.

## 2. Results and Discussion

### 2.1. Structure Changes of Modified Productions of BSPI

#### 2.1.1. Determination of Amino Acid Composition of BSPI and Its Modification Products

From a theoretical viewpoint, BSPI cross-linking and glycosylation catalyzed by TG does not change the relative content of amino acids in BSPI. The relative contents of amino acids in BSPI and its modified products are shown in Table 1. According to the experimental results, no significant decrease of lysine and arginine content of EGBSPI and ECBSPI is found, which indicates there was no significant Maillard reaction in the glycosylation for these two samples, while the content of lysine and arginine in WHTGBSPI decreased significantly, which suggests Maillard reactions had ocurred.

#### 2.1.2. SDS-PAGE Analysis of BSPI and Its Modification Products

**Figure 1:**
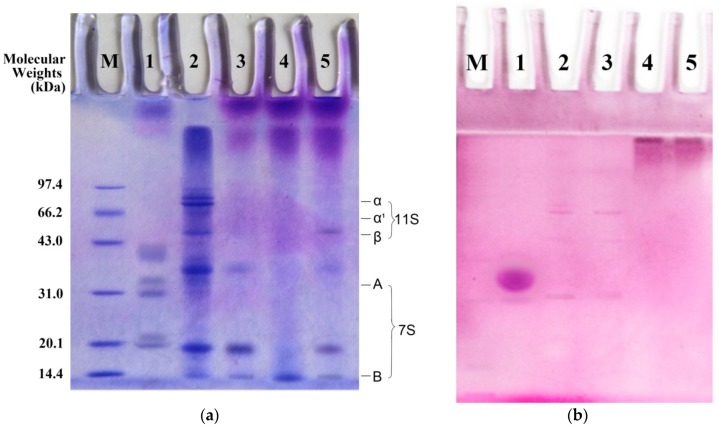
Electrophoretic profiles of analysis samples stained for protein (**a**) or saccharides (**b**). (**a**) protein staining; (**b**) glycocalyx stain. M represents standard protein marker; 1 represents horseradish peroxidase; 2 represents BSPI; 3 represents ECBSPI; 4 represents WHTGBSPI; 5 represents EGBSPI.

According to Figure 1a, compared to BSPI the protein subunit bands such as 7S and 11S globulins of ECBSPI, EGBSPI and WHTGBSPI 7S, 11S globulins are significantly reduced. During protein crosslinking catalyzed by TG, the free amino groups decreased, and the binding between Coomassie Brilliant Blue and protein molecules decreased, which results in the lighter staining color of subunit bands. At the same time, there are obvious spectrum bands in the concentrated gel, the junction of the concentrated gel and the separation gel, indicating the formation of protein copolymers. According to Figure 1b, there are obvious EGBSPI spectrum bands at the junction of the concentrated gel and the separation gel. Unlike ECBSPI and WHTGBSPI, two bands exhibit a characteristic glycocalyx stain, which is the same as the positive control horseradish peroxidase. This indicates that these two modified products contain glycosyl groups. Both the enzymatic and wet heat methods could make glycosylate BSPI and crosslink them with COS to form protein copolymer-glycoproteins.

#### 2.1.3. FT-IR Spectroscopy Analysis of BSPI and Its Modified Products

Each molecule had a unique FT-IR spectrum which is determined by its composition and structure. Accordingly, in this experiment, the side chain structure changes of the modified BSPI products were analyzed using FT-IR. The potassium bromide disk technique was used in the FT-IR spectroscopy analysis. 

**Figure 2:**
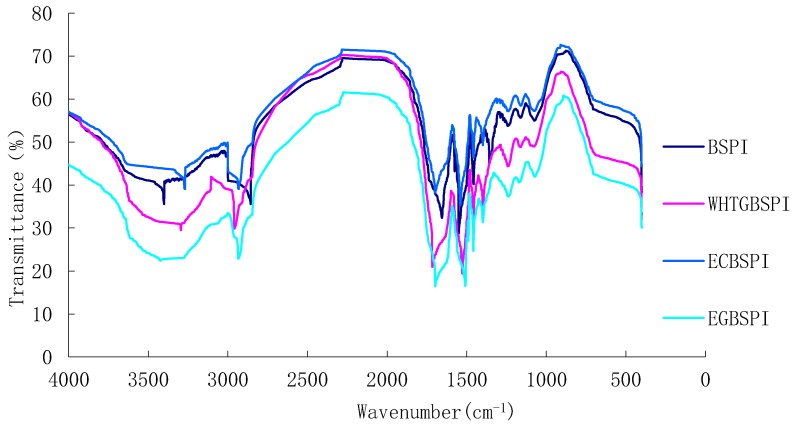
Comparison of FT-IR spectra of BSPI and its modified products.

As the results in Figure 2 show, compared with BSPI and ECBSPI, there are vibration expansions at 3365 cm^−1^ and 1070 cm^−1^ in EGBSP and WHTGBSPI, especially for a strong absorption peak at 1070.9 cm^−1^. When a protein covalently binds to a sugar molecule, a typical feature is an increase of the hydroxyl group (-OH) content in the protein molecule. The absorption peaks of 3700 cm^−1^~3200 cm^−1^ and 1260 cm^−1^~1000 cm^−1^ are found in the spectra, of which 1260 cm^−1^~1000 cm^−1^ is the stretching vibration region of C-C, C-O and the deformation region of O-H. The results further confirm that the TG catalyzed the reaction that connected COS to the BSPI with high efficiency.

#### 2.1.4. Circular Dichroism (CD) Spectroscopy Analysis of BSPI and Its Modified Products

**Figure 3:**
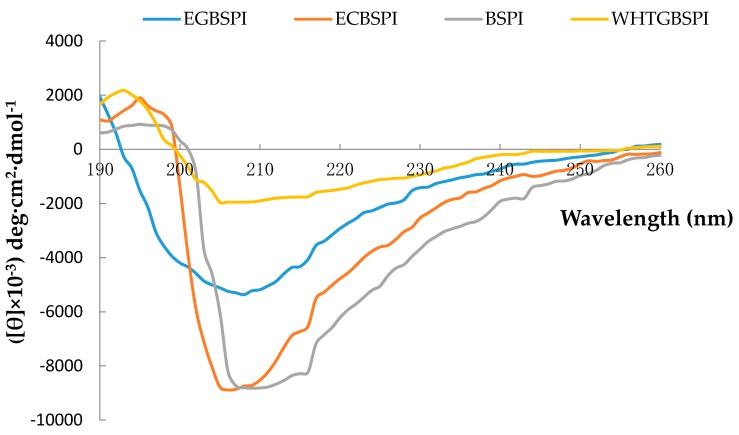
Circular dichroism spectra of BSPI and its modified products.

The secondary structural features of BSPI, ECBSPI, WHTGBSPI and EGBSPI were described by the CD spectra shown in Figure 3. According to the experimental results, BSPI shows a positive absorption around a wavelength of 195 nm and negative molar ellipticity around 216 nm which is a reflection of the β-sheet structure in the protein molecule. BSPI also show negative molar ellipticity at the wavelength of 208 nm which is a reflection of the α-helix structure in the protein molecule. All results suggest that the modified products have less α-helix and β-sheet structure, and possess a more open secondary structure than BSPI. BSPI is mainly composed of globulins with an ordered secondary structure [12]. Product modification by Maillard reaction and TG destroys parts of the α-helix and β-sheet structure to form BSPI with a relatively open secondary structure.

### 2.2. Structural Changes Characterized by the Properties of BSPI Modified Products

#### 2.2.1. Thermogravimetric Analysis of BSPI and Its Modified Products

**Figure 4:**
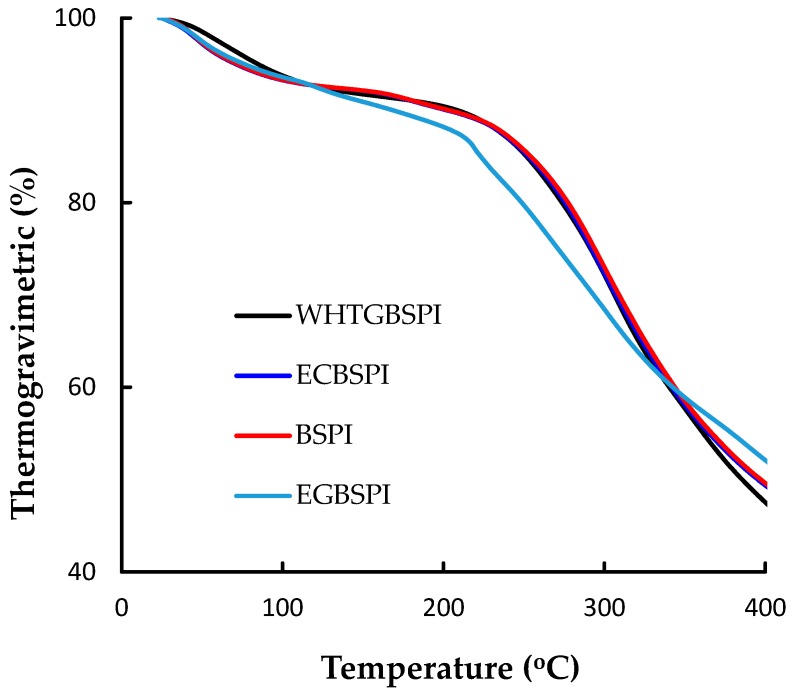
Thermogravimetric curves of BSPI and its modified products.

**Figure 5:**
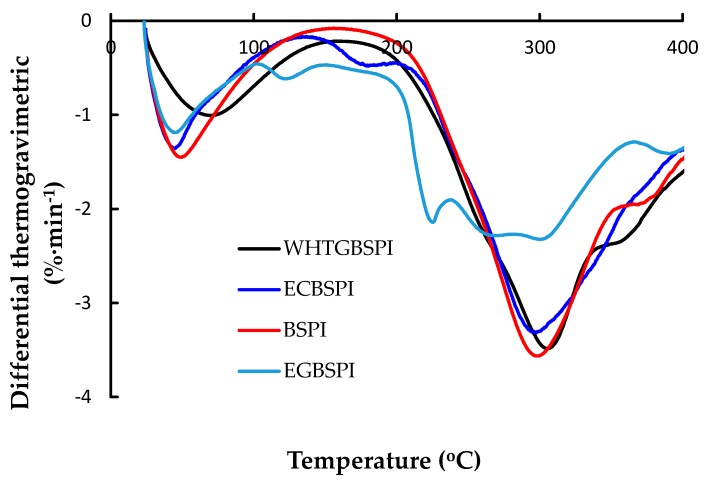
Differential thermogravimetric curves of BSPI and its modified products.

The thermal stabilities of BSPI, ECBSPI, WHTGBSPI and EGBSPI were determined by the classic thermogravimetric analysis technique using T_max_ and mass loss as the two indicators. The energy change of the heating treatment (ΔH) was calculated. Thermogravimetric and differential thermal gravity curves for the four samples are shown in Figure 4 and Figure 5. All samples have similar behavior during thermogravimetric analysis. There are two mass loss stages. Region I (ambient temperature to 105 °C): samples were heated at lower temperature without any covalent bond breakage. Mass loss of region I might mainly because of the water loss (either adsorbed or bound water). Compared with ECBSPI, WHTGBSPI and EGBSPI, BSPI loses water at a higher temperature with a lower amount of water loss. Region II (the temperature rose from 105 °C to 450 °C): samples partly decomposed. The measured T_max2_ of BSPI, ECBSPI, WHTGBSPI and EGBSPI are 297.3 °C, 290.7 °C, 286.4 °C and 292.1 °C, respectively, which suggest that WHTGBSPI has a less stable structure than BSPI, ECBSPI and WHTGBSPI. At the same time, the measured mass loss of BSPI, ECBSPI, WHTGBSPI and EGBSPI in region II are 45.6%, 46.5%, 48.1% and 42.6%, respectively. This confirms the thermal stability results of the samples [11]. During the whole heat treatment process, the total mass losses of BSPI, ECBSPI, WHTGBSPI and EGBSPI are 52.7%, 56.6%, 58.4% and 52.7%, respectively, and the energies (ΔH) of the thermal degradation reactions are 2.9, 2.6, 2.1 and 1.3 kJ/g. The decreasing trend of energy (ΔH) further confirms the thermal stability changes. In general, glycation leads to a decrease in ΔH, which is similar to the previous reported literature [13]. It could be summed up as the products of the TG method reaction have lower thermal stability than those of Maillard-type reactions, and both their thermal stabilities are inferior to BSPI. During the BSPI heat treatment process and its modified products, it mainly experienced the processes of water loss and thermal decomposition. The enthalpy value (ΔH), which measures the energy, is correlated with the ordered structure content according to the circular dichroism spectroscopy results [4], accordingly, the reduction of ΔH for modified BSPI compared to BSPI could be due to a decrease in the secondary structure content (α-helix, β-sheet) in BSPI or due to the disruption of BSPI intramolecular forces as a result of covalent conjugation with maltose.

#### 2.2.2. Zeta-Potential Analysis of BSPI and Its Modified Products

**Figure 6:**
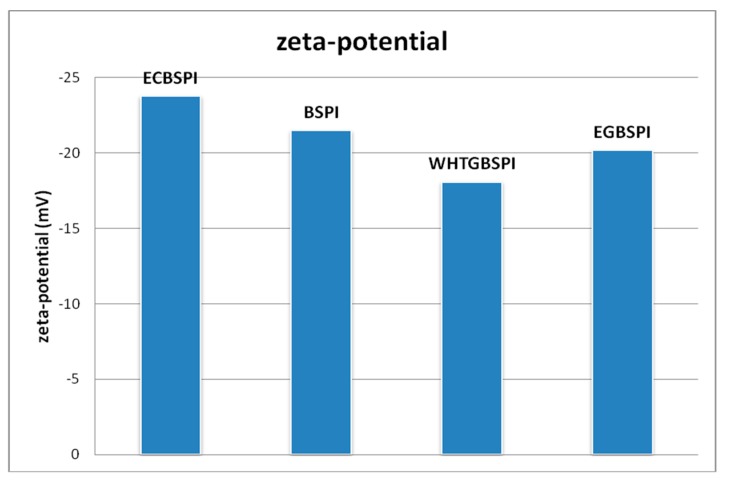
Zeta-potential of BSPI and its modified products.

As seen from Figure 6, the zeta-potentials of BSPI, ECBSPI, WHTGBSPI and EGBSPI are −21.5, −23.8, −18.1 and −20.2 mV, respectively. Wang et al. [14], reported that the β-potentials of whey protein isolate and a counterpart cross-linked by TG in dispersions were −27.0 mV and −30.4 mV, respectively. This conclusion is similar to the result for ECBSPI in this research. Li and Tang [15] found that glycation of BSPI by lactose through a Maillard-type reaction increased the zeta-potential of BSPI. Maillard-type reaction has the most negative zeta-potential, and would give the highest colloidal stability in dispersion. However there are some differences in our research. The increase of zeta-potential value of EGBSPI and WHBSPI might due to a neutralization reaction between the negative charge on the BSPI surface and positive charge on the COS surface.

#### 2.2.3. Surface Hydrophobicity Analysis of BSPI and Its Modified Products

According to the results shown in Table 2, compared to the surface hydrophobicity of BSPI, the surface hydrophobicity of EGBSPI and WHTGBSPI decrease, while the surface hydrophobicity of ECBSPI increases, which is similar to the report from Hiller [16]. The introduction of glycosyl groups could reduce the surface hydrophobicity due to the more hydroxyl groups in COS. Meanwhile, TG increased the surface hydrophobicity in the self-crosslinkages of BSPI, which is attributed to the hydrophobic amino acids embedded in protein molecules becoming exposed.

#### 2.2.4. Solubility of BSPI and Its Modified Products

**Figure 7:**
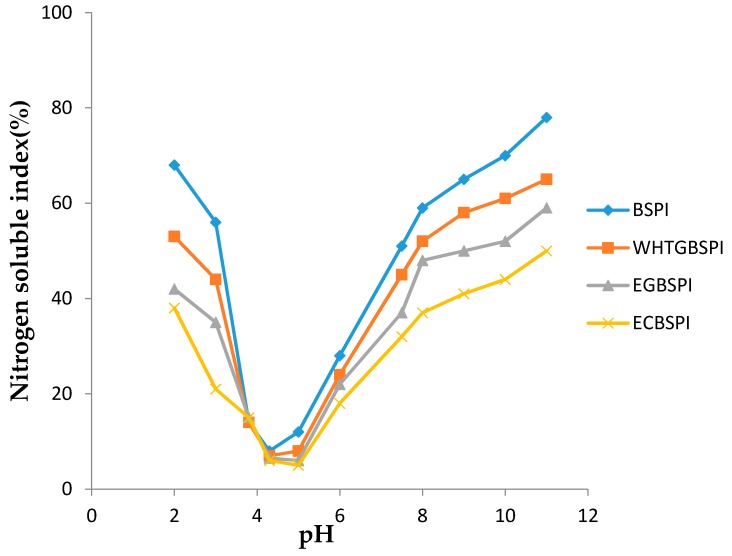
Solubility-pH profiles of BSPI and its modified products in pH range of 2~11. Data are shown as the mean ± SD of three replicates.

The solubility of BSPI and its modified production at pH 2~11 are shown in Figure 7. Among the four samples, BSPI has the best solubility while ECBSPI’s solubility is worst. This suggests that crossing-link of BSPI catalyzed by TG has an obvious influence on the solubility of BSPI. The significant decrease of protein solubility caused by crossing-link is because of the production of protein copolymer can be observed in the SDS-PAGE results. The solubility of EGBSPI is better than that of ECBSPI, and solubility of WHTGBSPI is better than both of them, owning to the introduction of glycosyls. Hydrophilic protein residues are embedded and hydrophilic hydroxyls are introduced into the protein which could enhance the overall hydrophilicity of the protein.

#### 2.2.5. Emulsification and Emulsion Stability of BSPI and Its Modified Products

**Figure 8:**
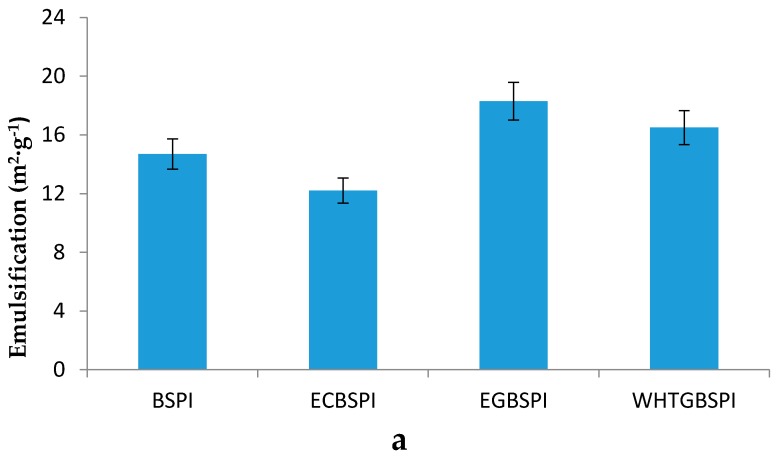

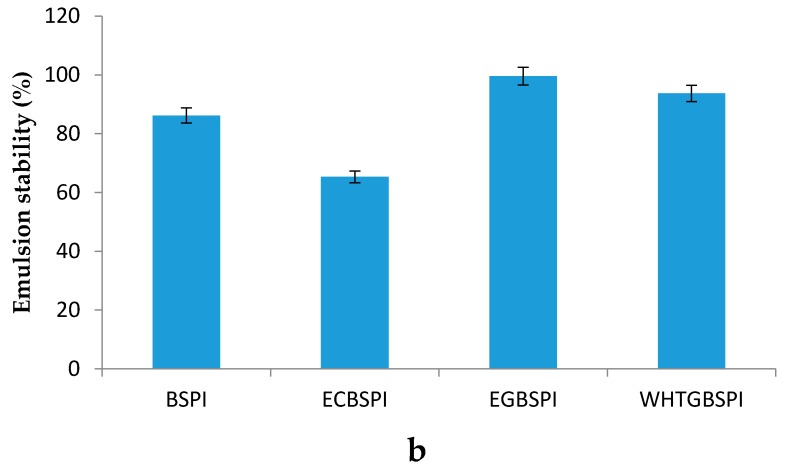
Emulsification (**a**) and emulsion stability (**b**) of BSPI and its modified products. Data are shown as the mean ± SD of three replicates.

The emulsification and emulsion stability of BSPI and its modified products are exhibited in Figure 8. The emulsion stability of samples changes with emulsification. EGBSPI has the best emulsification and emulsion stability while ECBSPI has the worst ones. According to Wooster and Augustin [13], the emulsion stability of proteins can be enhanced by glycosylation modification owning to the directional realignment of conjugated glycoprotein at the two-phase interface. Hydrophobic protein residues arrange towards the oil phase, while hydrophilic glycosyls arrange towards the water phase. The introduction of glycosyls into protein improves the surface hydrophilicity, which could impede the aggregation and condensation of oil drops, and this has a positive effect on the emulsion stability of proteins.

#### 2.2.6. Gelation Properties of BSPI and Its Modified Products

The textural properties of acid-induced gels prepared from BSPI and its modified products analysed by the TPA method are shown in Table 3. According to the data, EGBSPI exhibits similar textural properties to BSPI. Compared to BSPI, all the properties of WHTGBSPI decrease except adhesiveness, because the WHTGBSPI absorbed too much water during the gelling process. On the contrary, all the properties of ECBSPI increased except cohesiveness, owning to the crossing-link catalyzed by TG forming a more dense protein gel texture.

#### 2.2.7. Rheological Property of BSPI and Its Modified Products

**Figure 9:**
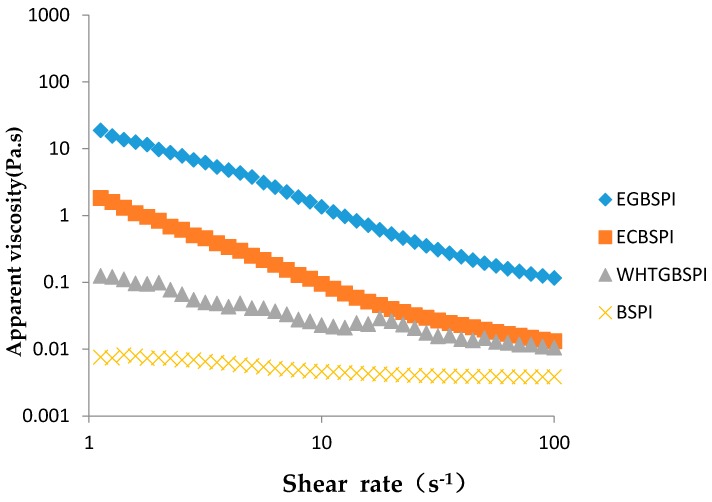
Rheological property of the suspensions prepared with BSPI and its modified products.

The rheological properties of BSPI and its modified products at the same mass fraction are described by the results shown in Figure 9. All four samples at 10% (*w*/*v*) concentration suspended liquid exhibit the shear thinning characteristics of non-Newtonian fluids. The apparent viscosity of all the suspended liquids of EGBSPI, ECBSPI and WHTGBSPI increased compared to those of BSPI, especially the modification catalyzed by TG. The introduction of the glycosyls leds to a raise of the apparent viscosity of the protein suspended liquids, owning to the hydrophilicity brought by glycosyls.

## 3. Materials and Methods

### 3.1. Materials

#### 3.1.1. Materials

Black soybean meal was kindly supplied by Jilin Baishibei Green Food Co., Ltd. (Jilin, China). COS (M = 2000 Da, degree of deacetylation 75%) was obtained from Zhejiang Golden Shell Pharmaceutical Co., Ltd. (Yuhuan, Zhejiang, China). TG was provided from Jiangsu Yiming Fine Chemical Industry Co., Ltd., (Taizhou, Jiangsu, China).

#### 3.1.2. Reagents

Spectroscopy grade KBr (Tianjin Jinbeier Tech. Co., Ltd., Tianjin, China), SDS-PAGE standard protein (Shanghai Institute of Biochemistry, Chinese Academy of Sciences, Shanghai, China), acrylamide (Sangong Biotech, Shanghai, China), methylene bisacrylamide (Sigma, St. Louis, MO, USA), trishydroxymethylaminomethane (Tris, Sigma), sodium dodecyl sulfonate (SDS, Sigma), ammonium persulfate (Sigma), Coomassie Brilliant Blue R-250 (Sigma), acetic acid (No. 3 Chemical Reagent Factory, Tianjin, China), methanol (Tian Da Reagent Factory, Tianjin, China), tetramethylethylenediamine (TEMED, Sinopharm Chemical Reagent Co., Ltd., Shanghai, China), horseradish peroxidase (HRP, Guo Yuan Biotechnology Co., Ltd., Shanghai, China), glucono-δ-lactone (Nanjing Chemical Reagent Co., Ltd., Nanjing, China). Unless stated otherwise all reagents were analytical purity quality.

#### 3.1.3. Instruments

H-1650 centrifuge (Xiangyi instrument Co., Ltd., Xiangtan, China), spectrum One FT-IR spectrometer (Perkin Elmer Inc., Norwalk, CT, USA), J-810 spectropolarimeter (Jasco Corporation, Tokyo, Japan), SDT Q600 TG analyzer (TA Instruments Inc., New Castle, DE, USA), dynamic light scattering (DLS) using the Zetasizer Nano-ZS90 (Malvern Instruments, Westborough, MA, USA), Discovery HR-1 rotary rheometer (TA Instruments Inc.), TA.XT.PLUS texture analysis (Stable Micro Systems, Godalming, UK), JJ-1/100Welectric agitator (Danrui Experimental Instrument Co., Ltd., Changzhou, China), PHS-3C Rex laboratory pH meter (Precision Scientific Instrument Co., Ltd., Shanghai, China), DYY-10C electrophoresis apparatus (Liuyi Instrument Factory, Beijing, China).

### 3.2. Preparation of BSPI and Its Modification Products

#### 3.2.1. Preparation of Black Soybean Protein Isolated (BSPI) [18]

Black soybean meal (100 g) was grounf to 40~60 mesh and extracted with 1.2 L water for 120 min (adjusting the pH to 8.0 with 1.0 mol/L NaOH) by stirring at 30~60 °C, 30~35 r·m^−1^. Then the extracts were centrifuged at 3000 r·m^−1^ for 10 min and supernatants were collected and adjusted to pH 4.4~4.6 with 1.0 mol·L^−1^ HCl (stirring until the isoelectric point). The extract liquid was allowed to stand for 30 min and the precipitate formed was collected and washed twice by diluted water. The protein was homogenized and pH adjusted to 6.5~7.0 with 5% NaOH and freeze-dried to give the BSPI sample. The dried sample was weighed and its amount was 34.32 g. The extraction rate of BSPI was 90.31% and the purity of the BSPI sample was 80.12%.

#### 3.2.2. Preparation of Enzymatic Glycosylation Black Soybean Protein Isolated (EGBSPI)

BSPI dispersion liquid (60 g·L^−1^) was mixed to prepare COS solution. TG solution was added (addition amount of 10 U·g^−1^ BSPI) to the mixture until the BSPI concentration in the reaction system reached 40 g·L^−1^ [18]. The molar ratio of acyl donors supplied by BSPI to acyl acceptors supplied by COS should be about 1:3 (each gram of BSPI could supply about 1 mmol of amide groups while each gram of COS can supply about 4.19 mmol of primary amine), the sample was glycosylated in a thermostatic vibration water bath at 37 °C for 3 h, and then the enzyme was inactivated by immersion ion a water bath at 85 °C. After cooling, the pH of the samples was adjusted to 4.5, and the samples centrifuged at 4000 r/m for 10 min [19]. The precipitate was collected and washed twice by diluted water, then it dialyzed at 4 °C and freeze dried for storage [20]. This is the optimal condition for making EGBSPI and the modification of EGBSPI was 15.16 g·kg^−1^ (glucosamine).

#### 3.2.3. Preparation of Enzymatic Crossing-Linked Black Soybean Protein Isolated (ECBSPI)

TG solution was added (10 U·g^−1^ BSPI) to the mixture until the BSPI concentration in the reaction system was 40 g·L^−1^. The cross-linking was carried out in a thermostatic vibration water bath at 37 °C for 3 h, and then the samples were placed in a water bath at 85 °C for enzyme inactivation. After cooling, the pH of the sample was adjusted to 4.5, and it was centrifuged at 4000 r·m^−1^ for 10 min. The precipitate was collected and washed twice by diluted water, dialyzed at 4 °C and then freeze dried for storage [20]. The same amount of TG was used in order to do the comparison.

#### 3.2.4. Preparation of Wet Heating Treatment Glycosylation Black Soybean Protein Isolated (WHTGBSPI)

BSPI dispersion liquid (60 g·L^−1^) was mixed to prepare COS solution. The molar ratio of acyl donors supplied by BSPI to acyl acceptors supplied by COS was about 1:3, and the glycosylation was carried in a thermostatic vibration water bath at 75 °C for 3 h, and then the samples were placed in a water bath at 25 °C for cooling down. After cooling, the pH of the sample were adjusted to 4.5, and it was centrifuged at 4000 r·m^−1^ for 10 min. The precipitate was collected and washed twice by diluted water, dialyzed at 4 °C and then freeze dried to store. The same amount of COS was used in order to do the comparison. The modification of EGBSPI is 18.10 g·kg^−1^ (glucosamine).

### 3.3. Methods

#### 3.3.1. Structure Changes of Modified Products of BSPI

(1) SDS-PAGE analysis of BSPI and its modified products

SDS-page and Coomassie Brilliant Blue staining were carried on according to Laemmli with some modifications [21]. The molecular mass of the SDS-PAGE standard proteins was as follows: phosphatase b (rabbit muscle), 97,400; bovine serum albumin (BSA), 66,200; egg albumin (egg white), 43,000; carbonic anhydrase, 31,000; trypsin inhibitor, 20,100; lysozyme (egg white), 14,400.

Two gels were prepared, one is for protein staining, the other one is for glycocalyx staining. The injection amount is 10 μL per track. The voltage is 80 V during the stacking gel. After the bromine blue front enters the separation gel, the voltage was adjusted to 120 V, and electrophoresis was ended when bromophenol blue was 0.5~1.0 cm from the lower edge.

(2) Determination of amino acid content of BSPI and its modified products

Determination of amino acid content was carried on according to GB/T 5009.124-2003. Exactly 0.05 g was weighed from each sample. Ten mL HCl at 6 mol/L and three drops of freshly distilled phenol were added to each hydrolysis tube. The hydrolysis tubes were kept in refrigerant for 5 min. The vacuum-nitrogen purge was repeated three times, and the hydrolysis tubes were kept in a thermostatic drying chamber at 110 °C for 22 h. All of the hydrolysate was removed and the volume adjusted to 50 mL in a volumetric flask. Filtrate (1 mL) was removed and made up to volume in a 5 mL volumetric flask and dried in thermostatic drier at 45 °C. The residues were dissolved with 2 mL water and dried twice. The sample solution was dried out and dissolved in 1 mL buffer (pH 2.2). Exactly 0.200 mL of mixed amino acid standard solution was diluted to 5 mL with buffer (pH 2.2). The amino acid contents of sample solution was detected by an automatic amino acid analyzer using the external standard method and calculated as shown in the Equation (1) below:

(1) $$X=\frac{c\times \frac{1}{50\;}\times F\times V\times M}{m\times 10^{9}}\times 100$$

where *X* represents the amino acid content of sample, unit: g·100 g^−1^, *c* represents the amino acid content of sample’s test solution, unit: nmol·50 μL^−1^, *F* represents the dilution multiple, *V* represents the constant volume of sample after hydrolysis, unit: mL, *M* represents the molecular weight of amino acid; *m* represents the sample mass, unit: g, $\frac{1}{50}$ represents the amino acid content determined after the sample was converted to each milliliter (unit: μmol·L^−1^), and $\;10^{9}$ represents the coefficient of ng converted to g.

(3) Fourier transform infrared spectroscopy (FTIR) analysis of BSPI and its modified products

According to Sang et al. [22], a potassium bromide pressing troche was used for FTIR. Potassium bromide (200 mg) was added to a 2 mg sample. The mixture was ground with a mortar and pressed into a thin slice. All band scanning of infrared spectrum (400~4000 cm^−1^) was taken and 32 scans were taken.

(4) Circular dichroism analysis of BSPI and its modified products

Far-UV circular dichroism (CD) can reflect the secondary structure of proteins according to a report of Wooster and Augustin [14]. Sample solutions (0.1 g·L^−1^ in 10 mmol L^−1^ phosphate buffer, pH 7.0) were tested at 25 °C. The experimental condition was as follows: spectra were recorded using a 0.1 cm path length cuvette; at the speed of 100 nm min^−1^; response time was 0.125 s and bandwidth was 1 nm. The results were shown as mean residue ellipticity (unit: deg·cm^2^·dmol^−1^).

#### 3.3.2. Structure Changes Characterized by Properties of BSPI Modified Productions

(1) Thermogravimetric analysis of BSPI and its modified products

In this experiment, dried samples were placed in a NaCl-saturated atmosphere for 24 h prior to analysis. Equilibrated samples (5 mg) were placed in open platinum pans, and s heated from 25 °C temperature to 450 °C at a ramp rate of 10 min^−1^, under a N_2_ flow fixed at 200 mL min^−1^. The temperatures responsible for the maximum degradation rates (T_max_) of the samples at two stages (25 °C~105 °C, and 105 °C~450 °C), as well as the mass loss of the samples, were determined from the derivative thermogravimetric (differential thermogravimetric) and thermogravimetric curves, respectively. The change of energy during heating treatment (ΔH) was also calculated [23].

(2) The measurement of zeta-potential analysis of BSPI and its modified products

Zeta-potential of samples (1.0 g L^−1^ in 10 mmol L^−1^ phosphate buffer, pH 7.0) of 1 mL which were prepared to a protein solution at 0.2% (mass fraction) detected at 25 °C.

(3) Surface hydrophobicity analysis of of BSPI and its modified products

Surface hydrophobicity was assayed according to Shigeru [24] with an ANS probe. The sample solutions were prepared at 0.05–0.5 g/L (on protein base) using 0.01 M phosphate buffer (pH 7.0). Aliquots (20 μL) of ANS solution (8.0 mmol L^−1^ in the same buffer) were added to 4 mL sample solutions. The fluorescence intensities of the solutions were measured at wavelength of 390 nm (excitation) and 470 nm (emission). The linear slope of the fluorescence intensity and protein content plot were regressed as an index of the surface hydrophobicity of the evaluated sample.

(4) Solubility of BSPI and its modified products

Certain amounts of protein samples were added into buffer at pH 2~11, vortexed and kept at 4 °C for 12 h. Supernatant was obtained after centrifugation at 8500 rpm for 20 min. Absorption values of samples at 280 nm were detected by an ultraviolet spectrophotometer. The buffer was prepared as follows: pH 2~3, 0.05 mol·L^−1^ citrate buffer, pH 4~5, 0.05 mol·L^−1^ acetate buffer, pH 6~8, 0.05 mol·L^−1^ phosphate buffer and pH 9~11, 0.05 mol·L^−1^ carbonate buffer.

(5) Emulsification and emulsion stability of BSPI and its modified products

Emulsification and emulsion stability of samples was evaluated according to the method of Pearce and Kinsella [25].

(6) Gelation of BSPI and its modified products

The gelation of samples was analyzed by texture profile analysis (TPA) of their acid-induced gels by a texture analyzer. The acid-induced gels of samples were prepared according to the method of Campbell et al. [26]. The acid-induced gel was kept at room temperature for 1 h and fixed on the measuring platform. Twice-compression method was chosen with the following parameters: probe p/36R, strain 50% and compression speed 1 mm·s^−1^. The main indices (hardness, adhesiveness, springiness, gumminess and chewiness) of this test were defined according to Song et al. [27].

(7) Rheological property of BSPI and its modified products

The samples were prepared as a liquid dispersion with pH 7.0 and 60 g·L^−1^ concentration. Plate geometry with 60 mm diameter was used. The apparent viscosity (η) of samples at the shear rate of 0.1~100 S^−1^ was detected.

#### 3.3.3. Statistical Analysis

The experimental data was analyzed by MS Excel 2007 (Microsoft Corporation, Redmond, WA, USA), and Origin 8.0 (Origin Lab, Northampton, MA, USA) was used to calculate or report the data.

## 4. Conclusions

According to the results, modified BSPI products are confirmed to be protein polymers. EGBSP and WHTGBSPI are glycation products, and there are glycoside bonds which suggests that COS are covalently bound to BSPI in them. Product modification by Maillard reactions and TG destroys parts of the α-helix and β-sheet structure to form BSPI with an open secondary structure. For ECBSPI, the proportion of irregular structure is reduced and the secondary structure is more orderly.

The surface hydrophobicity of EGBSP and WHTGBSPI decreased, while the surface hydrophobicity of ECBSPI increased. All the modified products could form greater aggregation in dispersion, but only WHTGBSPI has molecular stability. The solubility of EGBSPI becomes better than that of ECBSPI and the solubility of WHTGBSPI becomes better than both of them. Hydrophilic protein residues are embedded and hydrophilic hydroxyls are introduced into the protein which could enhance the overall hydrophilicity of the protein. All the textural properties of WHTGBSPI decreased except adhesiveness compared to BSPI. On the contrary, all the properties of ECBSPI increased except cohesiveness compared to BSPI, which might be due to the crossing-link catalyzed by TG making a more dense protein gel texture. As for the rheology, the apparent viscosity of all the suspended liquids of EGBSPI, ECBSPI and WHTGBSPI was increased compared to that of BSPI, especially the modification catalyzed by TG. Besides, the introduction of the glycosyls leads to an increase of the apparent viscosity of the protein-suspended liquids, which mighr be due to the hydrophilicity produced by the glycosyls.

In conclusion, most of the properties exhibited by EGBSPI are excellent, and the modification is efficient, and energy saving. Glycosylation of BSPI catalyzed by TG could be considered as a useful new modification technology in the food industry.

## Figures and Tables

**Table 1:** Relative content of the amino acids of BSPI and its modified products.

Amino Acid	BSPI (%)	EGBSPI (%)	ECBSPI (%)	WHTGBSPI (%)
Asp	8.4 ± 0.3	8.1 ± 0.2	8.7 ± 0.3	7.4 ± 0.2
Thr	2.2 ± 0.1	2.2 ± 0.1	2.3 ± 0.1	2.0 ± 0.1
Ser	3.5 ± 0.1	3.4 ± 0.1	3.7 ± 0.1	3.0 ± 0.1
Glu	16 ± 0.3	15 ± 0.4	16 ± 0.4	13 ± 0.5
Gly	2.7 ± 0.1	2.7 ± 0.1	2.9 ± 0.1	2.4 ± 0.1
Ala	2.7 ± 0.1	2.6 ± 0.1	2.9 ± 0.1	2.3 ± 0.1
Cys	0.9 ± 0.0	0.9 ± 0.0	0.9 ± 0.0	1.1 ± 0.0
Val	2.5 ± 0.1	2.4 ± 0.1	2.5 ± 0.1	2.2 ± 0.1
Met	0.7 ± 0.0	0.6 ± 0.0	0.8 ± 0.0	0.6 ± 0.0
Ile	2.5 ± 0.1	2.4 ± 0.1	2.5 ± 0.1	2.1 ± 0.1
Leu	4.9 ± 0.2	4.8 ± 0.1	5.1 ± 0.2	4.2 ± 0.1
Tyr	2.2 ± 0.1	2.0 ± 0.1	2.1 ± 0.1	1.8 ± 0.1
Phe	3.5 ± 0.1	3.4 ± 0.1	3.4 ± 0.1	2.9 ± 0.1
His	8.2 ± 0.2	8.8 ± 0.2	2.7 ± 0.1	6.8 ± 0.2
Lys	5.1 ± 0.1	4.5 ± 0.2	5.3 ± 0.2	4.3 ± 0.1
Arg	5.4 ± 0.1	4.9 ± 0.2	5.5 ± 0.2	4.5 ± 0.1
Pro	3.2 ± 0.1	2.8 ± 0.1	3.1 ± 0.1	2.4 ± 0.1

Data are shown as the mean ± SD of three replicates.

**Table 2:** Surface hydrophobicity evaluated results of BSPI and its modified products.

Protein Type	BSPI	ECBSPI	EGBSPI	WHTGBSPI
Surface hydrophobicity	16.8 ± 0.03	19.5 ± 0.06	5.07 ± 0.07	7.02 ± 0.05

Data are shown as the mean ± SD of three replicates.

**Table 3:** Textural properties of acid-induced gels prepared by BSPI and its modified products.

Textural Properties	BSPI	EGBSPI	ECBSPI	WHTGBSPI
Hardness (g)	167 ± 4.4 ^b^	148 ± 5.6 ^b^	380 ± 16.9 ^c^	71.4 ± 2.95 ^a^
Adhesiveness (g·s)	233 ± 11.4 ^a^	235 ± 10.7 ^a^	351 ± 13.0 ^b^	505 ± 21.8 ^c^
Springness	0.784 ± 0.039 ^a^	0.764 ± 0.034 ^a^	0.864 ± 0.039 ^b^	0.943 ± 0.046 ^c^
Cohesiveness	0.413 ± 0.016 ^c^	0.455 ± 0.018 ^c^	0.285 ± 0.013 ^b^	0.158 ± 0.006 ^a^
Guminess	69.1 ± 3.54 ^b^	67.2 ± 4.63 ^b^	108.3 ± 3.15 ^c^	11.3 ± 0.43 ^a^
Chewness	54.2 ± 1.37 ^b^	59.0 ± 2.95 ^b^	93.6 ± 3.56 ^c^	10.6 ± 1.63 ^a^

Data are shown as the mean ± SD of five replicates. Values that are marked with different letters in the same column are significantly different (*p* < 0.05).
